# Analysis of a Multi-Sector Regional Coalition to Combat Older Adult Isolation in a Rural U.S. County

**DOI:** 10.1093/geroni/igaf122.3704

**Published:** 2025-12-31

**Authors:** Michael Castellano, Lindsey Skripka, Christine Marcos, Lenard Kaye

**Affiliations:** United Way of Lackawanna, Wayne and Pike, Scranton, Pennsylvania, United States; Meals on Wheels of Northeastern Pennsylvania, Scranton, Pennsylvania, United States; Moses Taylor Foundation, Scranton, Pennsylvania, United States; University of Maine, orono, Maine, United States

## Abstract

Older adults residing in rural communities are at high risk of becoming socially isolated. The traditional scarcity of social and other supportive services in such settings often results in delays in effective community response. With Moses Taylor Foundation funding, a community collaborative of more than 30 health/human service, spiritual, philanthropic, and public agencies have created a formalized multi-sector organizational coalition to address older adult isolation in largely rural Lackawanna County, in northeastern Pennsylvania. Utilizing a collective impact model, a centralized infrastructure with its hub at the United Way of Lackawanna, Wayne, & Pike (UWLWP) has been successfully established. The Age Friendly Lackawanna Collaborative’s (AFLC) key components include dedicated staff, volunteer peer navigators, a common goal, shared measurement and data collection protocols, and coordinated, reframed aging communication channels. Key programmatic components that have been established, including screening, navigation, and awareness, ensure identification, guiding, and campaigning functions are performed, respectively. The AFLC has registered more than 167,000 Facebook views and 2,400 hits on the website in the first 6.5 months of 2025. Administering the evidence-based Wilder Collaboration Factors Inventory, confirms that a large majority of organizational partners (71% to 100%) concur that the seven (7) inventory factors aligned with the collective impact model have been successfully implemented. Strongest factor scores confirm achievement of an appropriate cross-section of members (90%) and shared membership stake in both process and outcome (100%). Best practice recommendations for replicating this collective community impact model to combat older adult social isolation in other rural communities are offered.

